# Problem and pro-social behavior among Nigerian children with intellectual disability: the implication for developing policy for school based mental health programs

**DOI:** 10.1186/1824-7288-36-37

**Published:** 2010-05-13

**Authors:** Muideen O Bakare, Vincent N Ubochi, Peter O Ebigbo, Andrew O Orovwigho

**Affiliations:** 1 Child and Adolescent Unit, Federal Neuro-Psychiatric Hospital, New Haven, Enugu, Enugu State, Nigeria; 2 Department of Psychological Medicine, University of Nigeria Teaching Hospital, (UNTH), Enugu, Enugu State, Nigeria

## Abstract

**Background:**

School based mental health programs are absent in most educational institutions for intellectually disabled children and adolescents in Nigeria and co-morbid behavioral problems often complicate intellectual disability in children and adolescents receiving special education instructions. Little is known about prevalence and pattern of behavioral problems existing co-morbidly among sub-Saharan African children with intellectual disability. This study assessed the prevalence and pattern of behavioral problems among Nigerian children with intellectual disability and also the associated factors.

**Method:**

Teachers' rated Strengths and Difficulties Questionnaire (SDQ) was used to screen for behavioral problems among children with intellectual disability in a special education facility in south eastern Nigeria. Socio-demographic questionnaire was used to obtain socio-demographic information of the children.

**Results:**

A total of forty four (44) children with intellectual disability were involved in the study. Twenty one (47.7%) of the children were classified as having behavioral problems in the borderline and abnormal categories on total difficulties clinical scale of SDQ using the cut-off point recommended by Goodman. Mild mental retardation as compared to moderate, severe and profound retardation was associated with highest total difficulties mean score. Males were more likely to exhibit conduct and hyperactivity behavioral problems compared to the females. The inter-clinical scales correlations of teachers' rated SDQ in the studied population also showed good internal consistency (Cronbach Alpha = 0.63).

**Conclusion:**

Significant behavioral problems occur co-morbidly among Nigerian children with intellectual disability receiving special education instructions and this could impact negatively on educational learning and other areas of functioning. There is an urgent need for establishing school-based mental health program and appropriate screening measure in this environment. These would afford early identification of intellectually disabled children with behavioral problems and appropriate referral for clinical evaluation and interventions. The need to focus policy making attention on hidden burden of intellectual disability in sub-Saharan African children is essential.

## Background

School based mental health programs are lacking in most Nigerian regular and special education institutions for children and adolescents and the burden of intellectual disability in sub-Saharan African children has largely been overlooked. Intellectual disability among children and adolescents is often associated with co-morbid behavioral problems [1-4]. These behavioral problems often influence the children's ability to cope with special education instructions and other areas of functioning. Co-morbid behavioral problems associated with intellectual disability, which often develop during childhood period, had been known to persist into the adulthood in most cases [5,6].

Studies addressing the issue of co-morbid behavioral problems that are often associated with intellectual disability and mental health interventions in children and adolescents are often lacking in Nigeria and other sub-Saharan African countries. Institutions offering special education facilities for children with intellectual disability are also limited and those few institutions in existence often do not have school based mental health program established.

Very few studies had addressed the issue of behavioral problems associated with intellectual disability among children in sub-Saharan Africa. In a school based study in Cape Town, South Africa [7], a high prevalence of thirty one percent psychopathology was recorded among children with intellectual disability. Molteno et al [7] found more behavioral problems in boys compared to girls and noted that children with severe and profound levels of intellectual disability showed more behavioral difficulties compared to those with mild and moderate degree of intellectual disability. In another school based study carried out in rural Western Cape Province of South Africa [8], thirty six percent of the school children studied were found to have one or more behavioral problems that may be clinically significant.

To the best of our knowledge, there has been no study in Nigeria that looked into behavioral problems that may be present co-morbidly with intellectual disability in school children.

This study therefore determined the prevalence and pattern of behavioral problems among Nigerian children with intellectual disability in a privately owned special education facility in south-eastern Nigeria. It also related behavioral problems to various socio-demographic variables of the children.

## Methods

### Location and participants

The location of the study was the Therapeutic Care Center (TCC), Abakpa, Enugu, Nigeria. This privately owned center was established to provide special education instructions, and behavioral modification to children with intellectual disabilities in south eastern Nigeria. The participants are children with intellectual disability admitted into the boarding facility of the TCC, Abakpa, Enugu, Nigeria. All children with suspected learning disability admitted into the center were studied. The children have associated co-morbid medical and psychological conditions ranging from Pervasive developmental disorders (PDD), Down syndrome and Cerebral palsy.

### Ethical consideration

Ethical approval for this study was obtained from the Institutional Review Board (IRB) of Federal Neuro-Psychiatric Hospital, New Haven, Enugu, Enugu State, Nigeria. Informed consent was obtained from the parents of the children studied and from the authority of Therapeutic Care Center (TCC), Abakpa, Enugu, Nigeria.

## Materials

### Socio-demographic questionnaire

This questionnaire was designed to obtain socio-demographic information such as age, gender among others, of the children studied.

### ICD - 10 diagnostic criteria for mental retardation [9]

The diagnostic criteria stated that depending upon the cultural norms and expectations of the individuals being studied; research workers must make their own judgments as to how best to estimate intelligence quotient (IQ) or mental age according to the following bands: profound mental retardation (IQ < 20, Mental Age < 3 years); severe mental retardation (IQ ≥ 20 ≤ 34, Mental Age = 3 -6 years); moderate mental retardation (IQ ≥ 35 ≤ 49, Mental Age = 6-8 years); mild mental retardation (IQ ≥ 50 < 70, Mental Age = 9 - 11 years).

Two clinicians that are of the same socio-cultural background as the children independently assessed each child with the diagnostic criteria in ICD - 10 and consensus was reached between them. Each child was allocated to specific categories of mental retardation, either as profound mental retardation, severe mental retardation, moderate mental retardation or mild mental retardation based on the clinicians' judgment.

The clinicians involved in the assessment of the children are psychiatrist trainees at senior registrar level of their training and had been working with children with intellectual disability at TCC, Abakpa, Enugu for a minimum period of six months. All assessments by the clinicians were based on the same specific socio-cultural milieu with the children. The procedure followed for IQ assessment of the children had been documented in our previous report [10]. This is an alternative method of IQ assessment which was employed because standardized tests of measuring IQ are lacking in sub-Saharan African children [10].

### Strengths and Difficulties Questionnaire (SDQ) [11,12]

SDQ is a brief and short screening instrument that can be used to screen for behavioral problems in children and adolescents. SDQ contains twenty five item questions and five clinical sub-scales of; Emotional Symptoms, Conduct Problems, Hyperactivity, Peer Problems and Pro-social Behavior. The sum of scores on scales of Emotional Symptoms, Conduct Problems, Hyperactivity and Peer Problems usually account for Total Difficulties score which can range from 0 to 40.

SDQ is designed in different versions to include;

Parent and Teacher rated version for children between ages 4 and 16 years

Self rated version designed for children between ages 11 and 16 years

There is also a supplement part of SDQ that enquires about chronicity, distress, social impairments and burden to others [12].

Strengths and Difficulties Questionnaire (SDQ) had been recommended as a useful screening tool for behavioral problems in children and adolescents [11]. SDQ had also been used in different population sample to screen for behavioral problems in children and adolescents and had been documented to have good psychometric properties [13]. These population samples included general population [14-17], regular school community [15], pediatric out-patients population [14] among others.

For the purpose of this study, the teacher rated version was used. The class teacher for each child rated the children on each item question of SDQ and total difficulties score for each child was obtained. Only the teachers rated the children on SDQ, parents rating were not obtained because the children involved in the study were in boarding facility of the Care Center and only have contacts with the parents during vacation periods and when the parents come visiting the school on social visits. We felt the teachers would give a more representative judgment of the children's assessment compared to the parents since they are more in contact with the children. The supplement part of SDQ that enquire about chronicity, distress, social impairments and burden to others were not included for teachers' rating.

Self rated SDQ was also avoided for those children 11 years old and above because of the influence we perceived intellectual disability might have on individual child's response to the item questions on SDQ.

### Procedure

The socio-demographic questionnaire was used to obtain demographic information of the children. Each child was assigned IQ category based on the clinicians' assessment and placement of the child in specific diagnostic categories as spelt out by ICD- 10 diagnostic criteria for mental retardation [9,10]. The teachers rated behavioral problems in the children by filling out the teacher's rated SDQ for each child.

The teachers involved in the assessment of the children had been providing routine services to the children at the TCC, Abakpa, Enugu, Nigeria for at least a period of six months. They were of the same socio-cultural background as the children, Igbo ethnic group in south eastern region of Nigeria. The scores in different clinical scales of SDQ were used to classify the studied children into abnormal, borderline and normal categories based on the cut off points recommended by Goodman [11].

### Data analysis

Data were analyzed using Statistical Package for Social Sciences (SPSS), version 16. Independent sample t-test was used to compare the mean scores of the children under total difficulties scale and pro-social scale of SDQ in different categories of mental retardation by combining the categories of mild to moderate as one group and the categories of severe to profound as another group. The gender disparity in behavioral problems as screened by SDQ was analyzed with t-test statistics and age influence on behavioral problem was analyzed with correlation statistics. The inter-clinical scales correlations of mean scores and internal consistency of SDQ in the population studied were also determined.

## Results

A total of forty four (44) children with intellectual disability participated in the study. The gender distributions according to different categories of mental retardation were as follows: profound (2 males and 0 female), severe (8 males and 3 females), moderate (15 males and 12 females), and mild (3 males and 1 female). In total, there are twenty eight (63.6%) males and sixteen (36.4%) females. Their age range was between 4 and 18 years, while the mean age was 13.2 ± 3.6 years. The score ranges and mean scores on total difficulties and other sub-scales of SDQ are: 0 to 7 and 2.95 ± 2.13 (emotional symptoms scale); 0 to 8 and 2.48 ± 2.04 (conduct problem scale); 0 to 9 and 4.93 ± 2.43 (hyperactivity scale); 0 to 8 and 3.09 ± 1.75 (peer problem scale); 0 to 10 and 6.57 ± 2.64 (pro-social scale); 2 to 27 and 13.68 ± 5.67 (total difficulties scale).

### Mental retardation categories based on ICD - 10 diagnostic criteria, total difficulties scores and pro-social scores on SDQ

Table 1 showed the different diagnostic categories of mental retardation in the children and their distribution. It also showed mean scores on total difficulties and pro-social scales of SDQ for each category of mental retardation. Children with intellectual disability in the category of mild mental retardation were more likely to have higher total difficulties score on SDQ compared to other categories. When the mean scores on total difficulties scale of SDQ were compared between the group of children with mild to moderate mental retardation (14.45 ± 5.73) and the group of children with severe to profound mental retardation (11.85 ± 5.27) using t-test statistics, there was no statistical significant difference in the two means (t = 1.41, df = 42, p = 0.17). Also, the difference in mean scores on pro-social scale between the two groups of mild to moderate categories (6.42 ± 2.81) and severe to profound categories (6.92 ± 2.25) did not show any statistical significant difference (t = - 0.57, df = 42, p = 0.57).

**Table 1:** Mental retardation categories based on ICD - 10 diagnostic criteria and total difficulties and pro-social scores on SDQ

Category	Mental Retardation	IQ Range	Median IQ	Mental Age Range (Years)	Frequency N (%)	Mean Total Difficulties Score on SDQ	Mean Prosocial Score on SDQ
**F 70**	Mild	50 - 70	60	9 - 11	4 (9.1)	18.25 ± 8.85	2.25 ± 1.71

**F 71**	Moderate	35 - 49	42	6 - 8	27 (61.4)	13.89 ± 5.13	7.04 ± 2.39

**F 72**	Severe	20 - 34	27	3 - 5	11 (25.0)	11.73 ± 5.67	6.64 ± 2.25

**F 73**	Profound	0 - 20	10	0 - 2	2 (4.5)	12.50 ± 3.53	8.50 ± 2.12

### Prevalence and pattern of behavioral problems in children with intellectual disability by teachers' rated SDQ

Based on various cut-off points recommended by Goodman [11] listed under Table 2, the children were classified into borderline and abnormal categories in different clinical scales of SDQ. Under the emotional symptoms clinical scale of SDQ, 15 (34.1%) of the children were classified as borderline and abnormal using a cut-off point of 5 to 10. Under the conduct problem clinical scale of SDQ, 19 (43.2%) of the children were classified as borderline and abnormal using a cut-off point of 3 to 10. Under the hyperactivity clinical scale of SDQ, 20 (45.5%) of the children were classified as borderline and abnormal based on cut-off point of 6 to 10. Under the peer problem clinical scale of SDQ, 25 (56.8%) of the children were classified as borderline and abnormal based on a cut-off point of 4 to 10. Twenty one (47.7%) of the children were classified as borderline and abnormal under total difficulties score clinical scale of SDQ based on cut-off point of 12 to 40. Sixteen (36.4%) of the children were classified under normal category in pro-social scale of SDQ based on cut-off point of 6 to 10 [11]. Table 2 showed classification of the children into different categories of normal, borderline and abnormal under different clinical scales of SDQ.

**Table 2:** Prevalence of behavioral problems in different clinical scales of SDQ

SDQ Scales	Abnormal N (%)	Borderline N (%)	Abnormal + Borderline N (%)	Normal N (%)	Total N (%)
**Emotional**	10 (22.7)	5 (11.4)	15 (34.1)	29 (65.9)	44 (100)
**Conduct Problem**	12 (27.3)	7 (15.9)	19 (43.2)	25 (56.8)	44 (100)
**Hyperactivity**	16 (36.4)	4 (9.1)	20 (45.5)	24 (54.5)	44 (100)
**Peer Problem**	16 (36.4)	9 (20.5)	25 (56.8)	19 (43.2)	44 (100)

**Total Difficulties Score**	13 (29.5)	8 (18.2)	21 (47.7)	23 (52.3)	44 (100)

**Pro-social**	18 (40.9)	10 (22.7)	28 (63.6)	16 (36.4)	44 (100)

Abnormal + Borderline Emotional Scale cut off points = 5 to 10

Abnormal + Borderline Conduct Problem Scale cut off points = 3 to 10

Abnormal + Borderline Hyperactivity Scale cut off points = 6 to 10

Abnormal + Borderline Peer Problem Scale cut off points = 4 to 10

Normal Pro-social Scale cut off points = 6 to 10

### Gender and behavioral problems

There were a total of twenty eight (63.6%) male and 16 (36.4%) female children with intellectual disability.

#### Emotional symptoms scale

Males and females had mean scores of 3.07 ± 2.18 and 2.75 ± 2.11 respectively on this scale of SDQ. There is no statistical significant difference in the mean scores (t = 0.47, df = 42, p = 0.64).

#### Conduct problem scale

Males and females had mean scores of 2.93 ± 2.23 and 1.69 ± 1.40 respectively on this scale of SDQ. The difference in mean scores between male and female reached a level of statistical significance (t = 2.00, df = 42, p = 0.05) with the males having a higher mean score.

#### Hyperactivity scale

Males and females had mean scores of 5.46 ± 2.50 and 4.00 ± 2.07 respectively on this scale of SDQ. The difference in the two mean scores was statistically significant (t = 1.98, df = 42, p = 0.05) with the males having a higher mean score.

#### Peer problem scale

Males and females had mean scores of 2.96 ± 1.58 and 3.31 ± 2.06 respectively on this scale of SDQ. The difference between the two mean scores was not statistically significant (t = - 0.63, df = 42, p = 0.53).

#### Total difficulties scale

Males and females had mean scores of 14.71 ± 5.08 and 11.88 ± 6.34 respectively on total difficulties scale of SDQ. Though higher mean score was found for male children with intellectual disability on this scale, the difference between the two mean scores was not statistically significant (t = 1.63 df = 42, p = 0.11).

The statistically significant difference in the mean scores on conduct and hyperactivity scales of SDQ indicated that children with intellectual disability in this study who are males were more likely to exhibit difficult behavior related to conduct and hyperactivity problems compared to their female counterparts.

#### Pro-social scale

Male and females had mean scores of 6.71 ± 2.64 and 6.31 ± 2.73 respectively on pro-social scale of SDQ. The difference between the two mean scores was not statistically significant (t = 0.48, df = 42, p = 0.63).

### Age and behavioral problems

Mean chronological age of the children with intellectual disability did not show significant correlation with mean scores on any clinical scale of SDQ. However, mean chronological age showed negative correlation with mean score on hyperactivity scale of SDQ that was almost approaching level of statistical significance (r = - 0.28, p = 0.06*). The implication of this is that the older the child, the lower the scores on difficult behavior related to hyperactivity. The correlations statistics between mean chronological age of the children with intellectual disability and mean scores on different clinical scales of SDQ are highlighted in Table 3.

**Table 3:** Correlation statistics between mean chronological age and mean scores on different clinical scales of SDQ

Emotional Symptoms Scale	Conduct Problem Scale	Hyperactivity Scale	Peer Problem Scale	Total Difficulties Scale	Prosocial Scale
r = 0.03 p = 0.83	r = 0.03 p = 0.86	r = -0.28 p = 0.06*	r = -0.05 p = 0.76	r = -0.18 p = 0.25	r = 0.01 p = 0.97

### Internal consistency and inter-scale correlations of different clinical scales of SDQ in the studied population

The inter-scale correlations of different clinical scales of SDQ among the studied population showed good internal consistency (Cronbach Alpha = 0.63). The mean score on pro-social scale was negatively correlated with mean score on total difficulties scale, but this did not reach a level of statistical significance (r = - 0.27, p = 0.08). Table 4 showed the inter-scale correlations of different clinical scales of SDQ.

**Table 4:** Inter-scale correlations of different clinical scales of SDQ

	Emotional Symptoms	Conduct Problem	Hyperactivity Scale	Peer Problem	Prosocial Scale	Total Difficulties
**Emotional Symptoms**		0.059	0.496	0.319	-0.115	0.691
**Conduct Problem**			0.320	0.190	0.074	0.540
**Hyperactivity Scale**				0.280	-0.286	0.816
**Peer Problem**					-0.418	0.582
**Prosocial Scale**						-0.265

**Figure 1:**
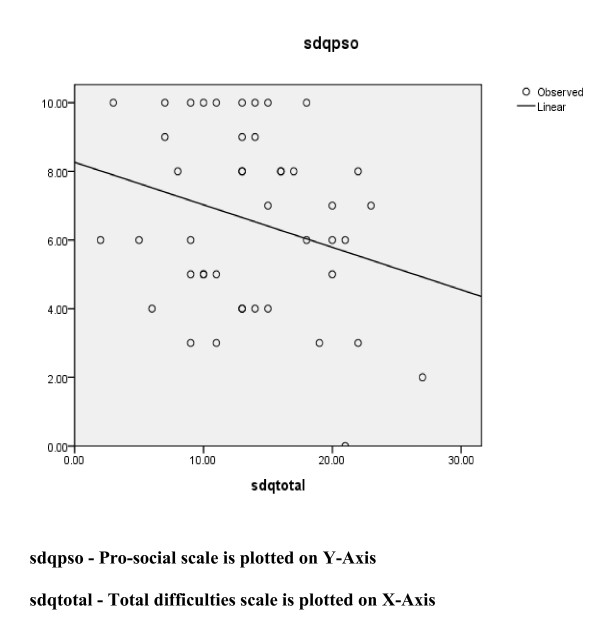
**Graphical relationship between SDQ pro-social scores and total difficulties scores**.

Figure 1 also showed the graphical relationship between pro-social scale scores and total difficulties scores for the studied population.

## Discussion

Behavioral problems had been identified as a significant issue among children and adolescents with intellectual disability. The prevalence of behavioral problems in children with intellectual disability documented by past studies carried out in developed parts of the world ranges from thirty one to forty one percent [2-5,16,17]. The findings in our own present study using teacher's SDQ rating showed a prevalence of about forty eight percent when the children classified under borderline and abnormal categories based on total difficulties cut-off score were combined. The prevalence of behavioral problems among children with intellectual disability in different clinical scales of SDQ ranges between thirty four and fifty seven percent, with emotional symptoms scale accounting for the lowest prevalence and peer problems scale accounting for the highest prevalence. Limited studies had been carried out in Nigeria and other sub-Saharan African countries that look into the issue of behavioral problems that may be coexisting with intellectual disability among children receiving special education instructions. Our finding is a reiteration of the observations that had been documented in previous studies carried out in developed regions of the world [2-5,7,8,18], and further establish that sub-Saharan African children with intellectual disability also experience co-morbid behavioral problems that would need adequate attention because it may impact negatively on educational learning and functioning in other areas and contribute to hidden burden of intellectual disability in these children.

Though male children with intellectual disability had higher mean score when compared to females on total difficulties scale of SDQ, this difference was not statistically significant. Male children with intellectual disability in this study were however likely to exhibit more conduct and hyperactivity behavioral problems when compared to the females. This finding is in contrast with that of Giarelli and colleagues [8] who found slightly higher females to males' proportion having psychopathology in their study. Our findings also differ from that of Einfeld and Tonge [2] who concluded that gender did not affect prevalence of psychopathology in their study. Our findings however concur with the findings of Molteno et al [7], who documented more behavioral problems in male than female children with intellectual disability in their study. The reasons for these variations are not certain, but may be due to differences in profile of children population studied or the different methodologies employed.

Chronological age did not show significant correlation with scores on different clinical scales of SDQ among the children with intellectual disability studied. The non influence of age on behavioral problems as documented in this study would be consistent with findings in previous longitudinal studies [5,6], that behavioral problems in children with intellectual disability often developed during childhood period and tend to persist into adulthood in most cases. However, chronological age showed a near significant negative correlation with scores on hyperactivity clinical scale of SDQ, indicating that behavioral problems relating to hyperactivity may be attenuated with increasing age of the children.

The need to establish school-based mental health programs in regular schools in Nigeria had earlier been identified [19]. Our findings in this study on significant level of behavioral problems co-existing with intellectual disability among children and adolescents receiving special education instructions in this environment further reiterate the urgency of establishing viable school-based mental health programs in both regular and special schools in Nigeria. Less attention had been focused on burden of intellectual disability among sub-Saharan African children when compared to the attention that had been given to burden of malaria and other communicable diseases. Mung'ala-Odera et al [20] had observed that significant burden of neuro-cognitive impairments including intellectual disability in Africa is likely to increase as more children of age five years and below continue to survive because of improved healthcare. Njenga [21] had also noted that aside the high prevalence of intellectual disability in Africa, discrimination and access to education and justice remained the major challenges facing individuals with intellectual disability in Africa. The findings of this study and these previous reports underscore the need for policy makers in sub-Saharan African countries to also focus attention on burden of intellectual disability on affected children and their caregivers in this environment.

### Limitation

The cut off points used on different clinical scales of SDQ were based on psychometric properties of SDQ as obtained in the western culture because there is no normative cut off points on SDQ clinical scales among Nigerian children as of now. This limitation however is not significant enough to impact on the social policy implication of the findings of this study, which is the need to encourage incorporation of school-based mental health programs into Nigerian regular and special education institutions for children and adolescents. Future studies in this environment would need to establish normative cut off points on SDQ clinical scales among Nigerian children.

## Conclusions

Nigerian regular and special education institutions for children and adolescents lack school-based mental health programs. The high prevalence of behavioral problems found among these children with intellectual disability undergoing special education training is a wakeup call for policy makers to encourage and establish school-based mental health programs in Nigerian schools. More policy-making attention also needs to be focused on the burden of intellectual disability among affected children and their care-givers in sub-Saharan Africa.

## Competing interests

The authors declare that they have no competing interests.

## Authors' contributions

All authors contributed to the conception of the study. MOB wrote the initial draft of the manuscript. MOB, VNU, POE and AOO were involved in revising the manuscript. All authors read and approved the final draft of the manuscript.
